# Chemical Constituents with Free-Radical-Scavenging Activities from the Stem of *Microcos paniculata*

**DOI:** 10.3390/molecules15085547

**Published:** 2010-08-12

**Authors:** Hua Fan, Guang-Zhong Yang, Tong Zheng, Zhi-Nan Mei, Xiang-Ming Liu, Yu Chen, Su Chen

**Affiliations:** 1 College of Pharmacy, South Central University for Nationalities, Wuhan 430074, China; 2 College of Biomedical Engineering, South Central University for Nationalities, Wuhan 430074, China; 3 College of Chemistry and Material Sciences, South Central University for Nationalities, Wuhan 430074, China

**Keywords:** *Microcos paniculata*, free-radical-scavenging activities, DPPH, ABTS, Co(II) EDTA-induced luminol chemiluminescence by flow injection

## Abstract

The free-radical-scavenging activities of various solvent extracts of *Microcos paniculata* were evaluated through *in vitro* model systems, such as 1,1-diphenyl-2-picrylhydrazyl (DPPH), 2,2'-azino-bis-(3-ethylbenzothiazoline-6-sulfonate) (ABTS) and Co (II) EDTA-induced luminol chemiluminescence by flow injection. In all three of these systems the ethyl acetate (EtOAc) extract showed the highest free-radical-scavenging activity compared with the other three (*n*-BuOH, water and petroleum ether) extracts. Free-radical-scavenging assay-guided chromatographic separation of the EtOAc extract, using a normal-phase and reverse-phase silica gel column chromatography yielded five compounds: a new triterpene named methyl 3*β*-*O*-*p*-hydroxy-*E*-cinnamoyloxy-2*α*,23-dihydroxyolean-12-en-28-oate (**1**), whose spectral data are presented for the first time, together with four known compounds, epicatechin (**2**), 3-*trans*-feruloyl maslinic acid (**3**), maslinic acid (**4**) and sucrose (**5**). All of the compounds were isolated from *Microcos paniculata* for the first time. The compounds were identified by spectroscopic methods. Among them, compound **2** displayed significant free-radical-scavenging activity which is similar to that of standard antioxidant ascorbic acid (V_C_) and therefore may be a promising natural antioxidant.

## 1. Introduction

The genus *Microcos*, belonging to the family Tiliaceae, comprises 60 species worldwide, spreading across African countries, India, Malaysia and Indonesia. In China, there are three species, including *Microcos paniculata* L., *Microcos chungii* (merr.) chun and *Microcos stauntoniana* G. don, which are mainly found across Southwest and West China [1].

*Microcos paniculata* L. is a shrub that is abundant in secondary forests and also grown as hedges. It has been used traditionally to prepare herbal medicines and traditional teas, while a limited number of reports concerning the chemical constituents and biological activities of *M. paniculata* have appeared in the literature. Previous phytochemical investigations revealed in *M. paniculata* the presence of flavones [2], triterpenes [3] and alkaloids [4,5], and it has been reported that the water extract of *M. paniculata* not only has significant analgesic effects [6], but that it also exerts beneficial pharmaceutical and preventative effects for coronary heart disease and angina pectoris [7]. The chloroform- and methanol-extract of *M. paniculata* could also act as a pesticide [8]. Based on previous research, it is evident that *M. paniculata* which contains a variety of active components has diverse pharmacological and dietary values.

Lipid peroxidation is one of the main factors causing deterioration of food products during storage and processing. Antioxidants play an important role in reducing nutritional losses and lengthening the shelf life of food, but the use of synthetic antioxidants, such as butylated hydroxyanisole (BHA) and butylated hydroxytoluene (BHT), which are widely used nowadays in processed food products, is now in doubt due to safety concerns about their potential toxicity and unwanted side effects [9,10]. Thus, more attention is increasingly being focused on the development and utilization of natural sources as antioxidants. To data, there are few reports addressing the evaluation of the free-radical-scavenging activities of the extracts and purified compounds from *M. paniculata*. Thus, it is necessary to conduct further research on the chemical constituents with free-radical-scavenging activities from this species.

## 2. Results and Discussion

### 2.1. Free-radical-scavenging activities of the solvent extracts of Microcos paniculata

**Figure 1:**
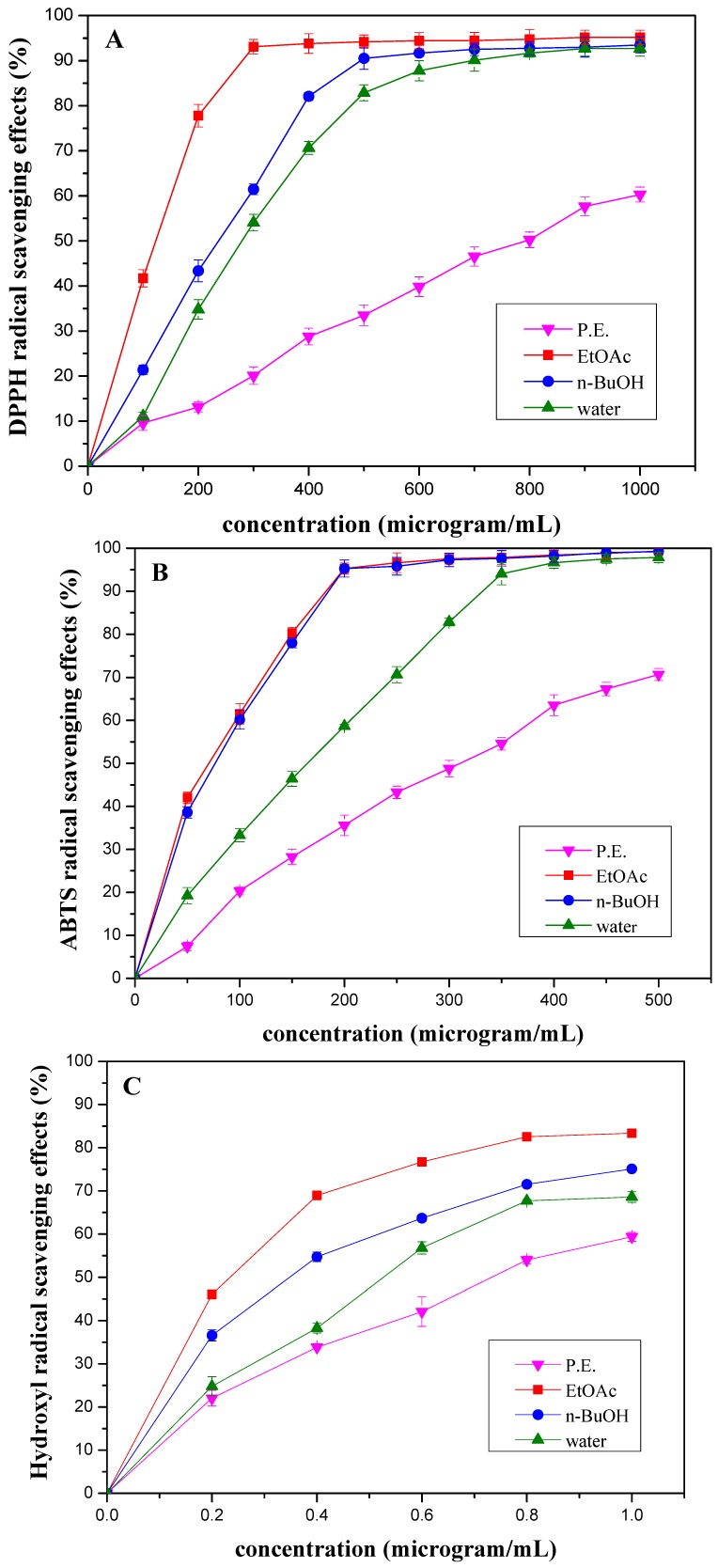
Scavenging activities of different concentrations of the extracts from *Microcos paniculata* towards **(A)** DPPH, **(B)** ABTS and **(C)** hydroxyl free radicals. Free-radical-scavenging activities were indicated as % inhibition. Each point was performed as mean ± standard deviation (SD) of three determinations.

Scavenging activities of the solvent extracts from *M. paniculata* towards DPPH, ABTS and hydroxyl radicals are shown in Figure 1, respectively. Figure 1 illustrates that the solvent extracts from the stems of *M. paniculata* showed a concentration-dependent DPPH radical scavenging activity. The DPPH radical scavenging effect of the solvent extracts at 500 μg/mL decreased as follows: EtOAc > *n*-BuOH > water > petroleum ether (P.E.), and were 94.19%, 90.50%, 82.85% and 33.48%, respectively (Figure 1A

The results of scavenging activities of the solvent extracts towards ABTS radical were in agreement with which obtained by the DPPH method. It is obvious that a significant decrease in the concentration of ABTS radial due to the scavenging ability of the solvent extracts. A 96.62% radical-scavenging activity by the EtOAc extract at 250 μg/mL was evident, whereas the *n*-BuOH and water extracts exhibited 95.80% and 70.65% inhibition of activities at the same concentration, respectively. The P.E. extract showed the lowest radical-scavenging activity (43.29% at 250 μg/mL) (Figure 1B). 

The results were found to be similar for the hydroxyl radical scavenging activities of the solvent extracts by Co (II) EDTA-induced luminol chemiluminescence by flow injection method. Our results indicated that the hydroxyl radical generation by luminol-H_2_O_2_-Co (II)-EDTA system was inhibited by the solvent extracts of *M. paniculata*. EtOAc, *n*-BuOH, water and P.E extracts led to an inhibition 76.71%, 63.68%, 56.79% and 42.08% at a concentration of 0.6 μg/mL, respectively (Figure 1C). Therefore, the EtOAc extract exerted the best hydroxyl radical scavenging effect. 

Based on the data obtained from this study, it was observed that the EtOAc extract was the most powerful free radical scavenger against DPPH, ABTS and hydroxyl radicals when compared with the other solvent extracts from *M. paniculata*, which was subjected to further separation by chromatographic methods to purify the active compounds, as illustrated in the following section.

### 2.2. Spectral analyses of the compounds isolated from Microcos paniculata

Compound **1** was obtained as a white power, whose molecular formula was determined as C_40_H_56_O_7_ by the HR-ESI-MS (found 671.3914 [M+Na]^+^, calcd. 671.3924). The ^1^H-NMR spectrum of **1** exhibited signals due to an AA'BB' type aromatic ring signal at *δ*_H_ 7.43 and 6.77 (each 2H, d, *J* = 8.4 Hz) and *trans* olefinic protons conjugated with an aromatic ring (*δ*_H_ 6.35 and 7.61, each 1H, d, *J* = 16 Hz) in which a proton at *β*–position to carbonyl group appeared in the lower field, suggesting the presence of a *p*-hydroxy-*E*-cinnamoyl moiety in **1**, which was supported by the ^13^C-NMR data. In addition to a *p*-hydroxy-*E*-cinnamoyl moiety, its ^1^H-NMR showed six tertiary methyls [δ 0.73 (3H, s), 0.75 (3H, s), 0.88 (3H, s), 0.90 (3H, s), 1.05 (3H, s), 1.16(3H, s)], two oxygenated methines [δ3.90 (1H, m), 4.89 (1H, br s)], and an olefinic proton [δ5.24 (1H, br s)]. The ^13^C-NMR and DEPT spectra of **1** exhibited signals for thirty carbons, including six methyls, ten methylenes (an oxygenated CH_2_ at *δ*_C_ 65.1), five methines (two oxygenated CH at *δ*_C_ 67.7 and 79.9), six quaternary carbons, a trisubstituted double bond (*δ*_C_ 123.6 and 146.7) and a ester group (*δ*_C_ 180.8), which were compatible with that of the △^12^-oleanene type skeleton. The ester substituent was placed at C-3 as a result of the downfield shift observed for H-3 (*δ*_H_ 4.89) in the ^1^H-NMR spectrum, compared with that of methyl arjunolate [11]. It was observed that the spectral data of **1** was quite similar to that of methyl 3*β*-*O*-(4''-*O*-methyl-*E*-coumaroyl)-arjunolate [11], the only significant difference in the ^1^H-NMR spectra being that the methoxy group at the C-4'' position in the latter was replaced in **1** by a hydroxyl group. Therefore, based on all the above evidence the structure of the new compound **1** was assigned as methyl 3*β*-*O*-*p*-hydroxy-*E*-cinnamoyloxy-2*α*, 23-dihydroxy-olean-12-en-28-oate.

**Figure 2:**
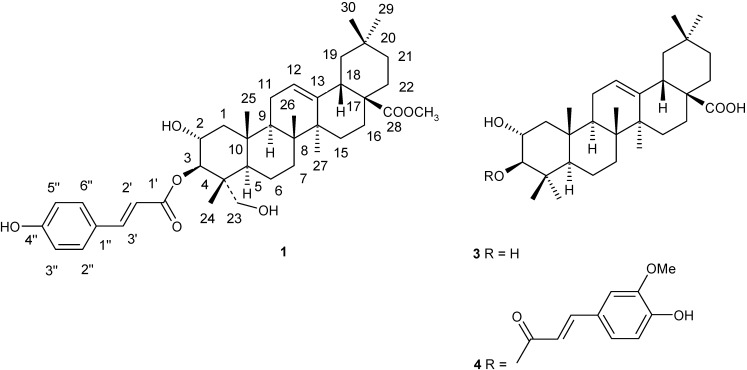
Structures of the compounds **(1, 3** and **4)** isolated from *Microcos paniculata*.

Compound **2** was obtained as a white power. Its spectral data agreed well with those of epicatechin [12]. Compound **3** was obtained as a white power. This compound was identified as 3-*trans*-feruloyl maslinic acid by comparing the obtained data with those reported in [13]. Compound **4** was obtained as a white power. Its spectral data were identical with the data reported for maslinic acid [14]. Compound **5** was obtained as a colorless crystalline block. The spectral data agreed well with the data reported in [15], so the compound was identified as sucrose. The structures of the compounds isolated from *Microcos paniculata* are shown in Figure 2. 

### 2.3. Free-radical-scavenging activities of the purified compounds of Microcos paniculata

**Figure 3:**
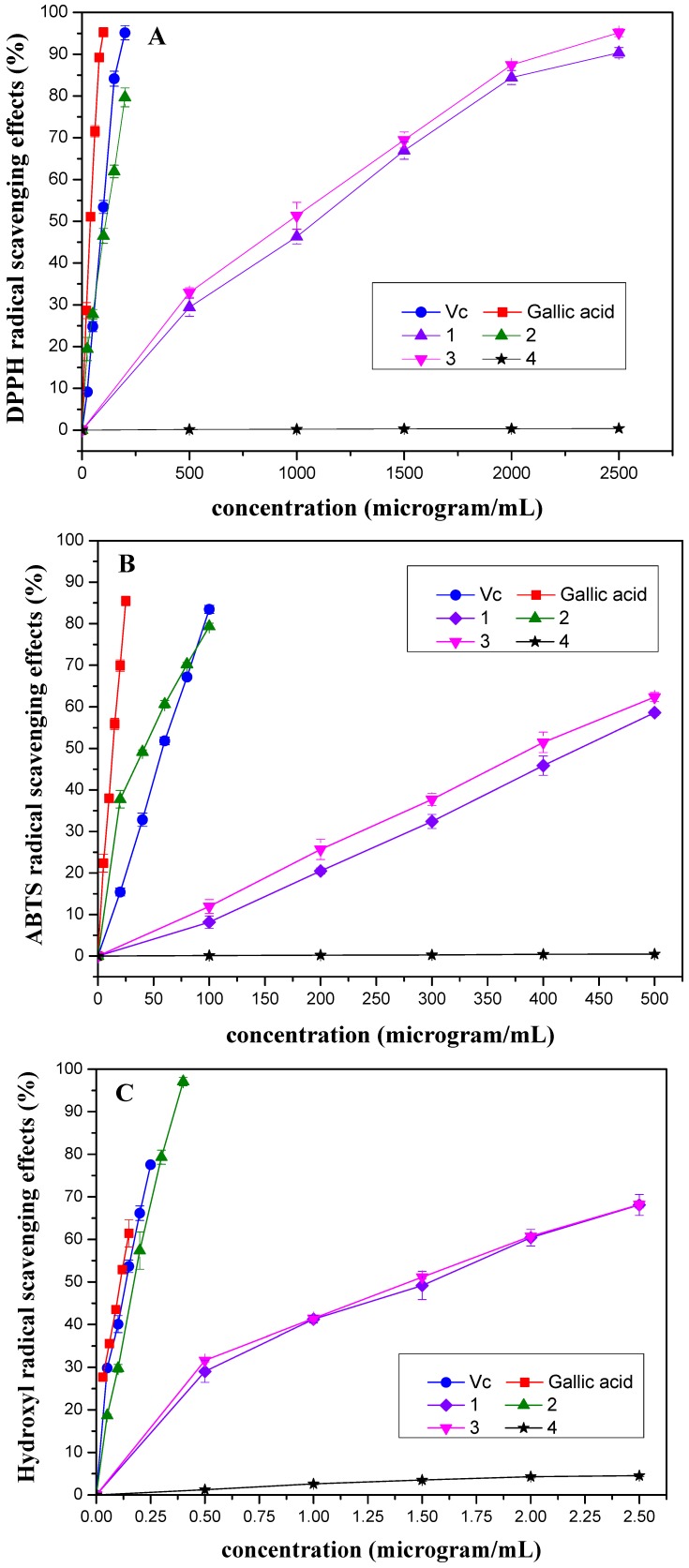
Scavenging activities of different concentrations of **1**-**4** isolated from *Microcos paniculata* towards **(A)** DPPH, **(B)** ABTS and **(C)** hydroxyl radicals. Free-radical-scavenging activities were indicated as % inhibition. Each point was performed as mean ± standard deviation (SD) of three determinations.

Scavenging activities of the purified compounds of *M. paniculata* towards DPPH, ABTS and hydroxyl radicals, respectively, are shown in Figure 3, which illustrates that the radical-scavenging effects were found to increase with the increasing concentration of the tested compounds. 

As shown in Table 1, The IC_50_ values of DPPH radical scavenging activity decreased as follows: **4** > **1** > **3** > **2**. DPPH radical is considered to be a stable free radical and used to evaluate the antioxidant activities of natural products in organic solvents. The DPPH method is based on the color decrease occurring when the odd electron of the nitrogen atom in DPPH radical is reduced by receiving a hydrogen atom from antioxidants. 

The ABTS assay is based on the antioxidant ability (in terms of radical-scavenging capacity) to react with ABTS^+^ generated in the system. The method is widely used to evaluate antioxidant activity in foods and biological systems and a high TEAC value indicated a high level of antioxidant activity [16]. From the TEAC values listed in Table 1, the ABTS radical scavenging activity of the tested compounds was in a decreasing order: **2** > **3** > **1** > **4**, which correlated highly with the results measured by the DPPH assay.

Scavenging activities of the purified compounds towards hydroxyl radical is measured by Co (II) EDTA-induced luminol chemiluminescence by flow injection method which based on the catalytic oxidation of hydrogen peroxide by luminol-H_2_O_2_-Co (II)-EDTA system, forming a hydroxyl radical flux that can produce a stable chemiluminescence signal which is attenuated in the presence of antioxidants. As shown in Figure 3C and Table 1, the hydroxyl radical scavenging activity of the tested compounds decreased in the order of: **2** > **3** > **1** > **4**, which was consistent with the results of the DPPH and ABTS assays.

It was observed that radical-scavenging activity of the purified compounds of *M. paniculata* decreased with the order: **2** > **3** > **1** > **4** in all three tests mentioned above, although the potency of the compounds was quite different in the three assays. Among the tested molecules, Compound **2**, with five hydroxyl groups (including four phenolic hydroxyl groups), exerted the highest free-radical-scavenging activity which is similar to that of standard antioxidant ascorbic acid (V_C_), one of the strongest antioxidants in the food. The presence of an *ortho*-dihydroxy group in the B-ring and a hydroxyl group at the 3-position of the C-ring is essential for the strong radical scavenging activity of **2** [17]. Compounds **1** and **3** which have 1 phenolic hydroxyl group exhibited almost the same scavenging capacity, which is significantly weaker than that of **2**. These results supported the idea that free-radical-scavenging activity can be attributed to the number of protons available for donation by free hydroxyl groups, and the phenolic hydroxyl structural group in benzene ring contributes much to the free-radical-scavenging activity [18,19]. This is further supported by the fact that compound **4** with only two hydroxyl groups (no phenolic hydroxyl groups) showed the lowest activity (not detected). 

**Table 1:** *In vitro* antioxidant activities of the purified compounds **(1-4)** from *Microcos paniculata.*

Compound	DPPH radical-scavenging effects (IC_50_. μg/mL )^a^	ABTS radical-scavenging effects (TEAC. μg/mL)^b^	Hydroxyl radical-scavenging effects (IC_50_. μg/mL )^a^
**1**	26.69 ± 1.33	0.13 ± 0.01	1.52 ± 0.04
**2**	2.83 ± 0.12	1.38 ± 0.05	0.18 ± 0.01
**3**	24.13 ± 1.23	0.15 ± 0.01	1.49 ± 0.01
**4**	ND^c^	ND^c^	ND^c^
**Ascorbic acid** **(V_C_** **)^d^**	2.31 ± 0.06	0.97 ± 0.02	0.13 ± 0.01
**Gallic acid^d^**	1.00 ± 0.03	4.13 ± 0.02	0.11 ± 0.01

^a^ IC_50_ (μg/mL) values were obtained by interpolation from linear regression analysis. Values were performed as means of three replicate determinations ± standard deviation (SD); ^b^ TEAC (μg /mL) values were performed as means of three replicate determinations ± standard deviation (SD). Values were expressed as μg trolox equivalents/μg tested compounds; ^c^ ND = not detected; ^d^ Ascorbic acid (V_C_) and gallic acid severed as positive controls, respectively.

## 3. Experimental

### 3.1. General

The ^1^H- and ^13^C-NMR spectra were measured on a Bruker-AM-400 NMR spectrometer at room temperature, using TMS as an internal standard. Chemical shifts (*δ*) are expressed in parts per million (ppm), with the coupling constants (*J*) reported in Hertz (Hz). The ESI-MS and HR-ESI-MS were recorded on Finnigan LCQ-Deca and Waters/Micromass Q-Tof-Ultima mass spectrometers, respectively. Column chromatographies were carried out with silica gel 60 H (200-300 mesh, Qingdao Haiyang Chemical Group Co., China), C_18_ reversed-phase silica gel (YMC Co., Ltd., Japan) and Sephadex LH-20 (Pharmacia), TLC was performed on Pre-coated silica gel GF_254_ plate (Qingdao Haiyang Chemical Group Co., China), with spots detected by UV_254_ and anisaldehyde/H_2_SO_4_ (10%). HPLC was performed on Ultimate 3000 HPLC system, and waters 5C_18_-MS-II (10 × 250 mm) column was used. 1,1-Diphenyl-2-picrylhydrazyl (DPPH), 2,2'-azino-bis(3-ethylbenzothiazoline-6-sulfonate) (ABTS) and 6-hydroxy-2,5,7,8-tetramethylchroman-2-carboxylic acid (Trolox) were obtained from Sigma-Aldrich. Gallic acid and ascorbic acid (V_C_) were purchased from the Chemical Company, Shanghai, P. R. China. Other chemicals and solvents in this experiment were all analytical grade or higher and from Shanghai Chemical Reagent Co.

### 3.2. Plant material

The stems of *Microcos paniculata* were collected from Xishuangbanna prefecture, Yunnan province, P. R. China and identified by the Xishuangbanna Prefecture National Medicine Research Institute. A voucher specimen was deposited in the Herbarium of the College of Pharmacy, South Central University for Nationalities. 

### 3.3. Extraction procedures

The dried and powered stems of *Microcos paniculata* (2.8 kg) were extracted three times at room temperature with 95% ethanol (EtOH, 5 L). The EtOH extract was evaporated under vacuum to dryness to afford a dark brown mass (261 g) and then the concentrated EtOH extract was suspended in 90% H_2_O/methanol (MeOH, 2 L). The solution was successively partitioned with P.E.(2.5 L), EtOAc(3L) and *n*-BuOH (4 L), to yield 8, 38, and 83 g of the corresponding extracts, respectively. 

### 3.4. Isolation procedures

The EtOAc extract (38 g) was chromatographed on silica gel with P.E.- acetone (Me_2_CO) (9:1, 8:2, 7:3, 6:4, 1:1, 4:6, 2:8, v/v) to provide nine fractions (Fr.1-Fr.9). Fr.3 (264.4 mg) was subjected to column chromatography (silica gel, cyclohexane/EtOAc 8:2, 7:3, 6:4, 4:6, 3:7, v/v) to give seven fractions (Fr.3.1-Fr.3.7), compound **4** (3 mg) was crystallized from Fr.3.4. Fr.4 (210.9 mg) was subjected to column chromatography (Sephadex LH-20, H_2_O/MeOH 0:1) to afford seven fractions (Fr4.1-Fr4.7). Fr.4.4 was purified by semi-prep. HPLC (MeOH/H_2_O 95:5, 2.0 mL/min) to obtain compound **3** (11.4 mg) at t_R_ 8.8min and compound **1** (4.6 mg) at t_R_ 9.7 min, respectively. Compound **2** (21.8 mg) was crystallized from Fr.8. Fr.9 (25.5 g) was subjected to column chromatography (ODS, H_2_O/MeOH 1:0, 9:1, 8:2, 7:3, 6:4, 5:5, 3:7) to give compound **5** (29.2 mg). 

### 3.5. Spectral data of compound ***1*** isolated from M. paniculata

^1^H-NMR (400 MHz, CD_3_OD): *δ*_H_ 0.73, 0.75, 0.88, 0.90, 1.05, 1.16 (each 3H, s, 6×CH_3_), 3.90 (1H, m, H-2), 4.89 (1H, br s, H-3), 5.24 (1H, br s, H-12),3.58 (3H, s, OCH_3_), 6.35 (1H, d, *J* = 16 Hz, H-2'), 7.61 (1H, d, *J* = 16 Hz, H-3'),7.43 (2H, d, *J* = 8.4 Hz, H-3''/5''), 6.77 (2H, d, *J* = 8.4Hz, H-4''/6''); ^13C^-NMR (100 MHz, CD_3_OD): *δ*_C_ 47.0 (C-1), 67.7 (C-2), 79.9 (C-3), 43.0 (C-4), 47.7 (C-5), 18.9 (C-6), 33.2 (C-7), 40.6 (C-8), 48.1 (C-9), 38.9 (C-10), 24.0 (C-11), 123.6 (C-12), 146.7 (C-13), 42.8 (C-14), 28.7 (C-15), 24.6 (C-16), 47.7 (C-17), 42.8 (C-18), 43.5 (C-19), 31.7 (C-20), 34.8 (C-21), 33.6 (C-22), 65.1 (C-23), 14.5 (C-24), 17.6 (C-25), 17.5 (C-26), 26.5 (C-27), 180.1 (C-28), 33.5 (C-29), 23.9 (C-30), 169.9 (C-1'), 115.5 (C-2'), 145.2 (C-3'), 133.8 (C-1''),131.2 (C-2''), 116.8 (C-3''), 161.3 (C-4''), 116.8 (C-5''), 131.2 (C-6''), 52.2 (28-OMe); ESI-MS: *m/z* 671 [M+Na]^+^; HRESI-MS: *m/z* 671.3914 [M+Na]^+^ (calcd for C_40_H_56_O_7_Na, 671.3924).

### 3.6. Free-radical-scavenging activities of the solvent extracts and purified compounds

#### 3.6.1. The free-radical-scavenging activities of the extracts and purified compounds were evaluated through 1,1-diphenyl-2-picrylhydrazyl (DPPH) method

Scavenging activities of the extracts and purified compounds from *M. paniculata* towards DPPH radical were assessed by using the method described by Scherer and Godoy with a slight modification [20,21]. Briefly, a 0.08 mM solution of DPPH radical solution in methanol was prepared and then, the solvent extracts and purified compounds at different concentrations (3.9 mL) were added to the prepared DPPH radical solution (0.1 mL); the mixture was shaken vigorously, after a 30 min incubation period at 37 ºC in the dark, the absorbance was measured at 517 nm by using a UV-visible spectrophotometer. Obviously, decreasing of the DPPH solution absorbance indicates an increase of the DPPH radical-scavenging activity. The radical scavenging activity is given as DPPH radical scavenging effect that is calculated using equation (1):

DPPH radical scavenging effect (%) = [(A_0_-A_1_)/A_0_] × 100 (1)

where A_0_ was the absorbance of control and A_1_ was the absorbance in the presence of the standard, solvent extracts or purified compounds at different concentrations. Ascorbic acid (V_C_) and gallic acid served as positive controls, respectively. All the tests were performed in triplicate. The scavenging activities of the purified compounds towards DPPH radical were expressed as IC_50_, which was determined to be effective concentration at which DPPH radical was scavenged by 50%. The IC_50_ value was obtained by interpolation from linear regression analysis.

#### 3.6.2. The free-radical-scavenging activities of the extracts and purified compounds were evaluated through 2,2'-azino-bis-(3-ethylbenzothiazoline-6-sulfonate) (ABTS) method

Scavenging activities of the extracts and purified compounds from *M. paniculata* towards ABTS radical were also measured [22,23,24]. Briefly, a stock solution of ABTS radical cation was prepared by dissolving ABTS (7 mM, 25 mL in deionised water) with potassium persulfate (K_2_S_2_O_8_) (140 mM, 440 μL). The mixture was left to stand in the dark at room temperature for 15-16 h (the time required for formation of the radical) before use. For the evaluation of ABTS radical scavenging activity, the working solution was prepared by the previous solution and diluting it in ethanol to obtain the absorbency of 0.700 ± 0.02 at 734 nm (ABTS working solution should be replaced every five days at least because the free radical degrades easily). The solvent extracts and purified compounds (0.1 mL) at different concentrations were mixed with the ABTS working solution (1.9 mL) and the reaction mixture was allowed to stand at 30 ºC for 6 min, then the absorbance was measured by using a UV-visible spectrophotometer at 734 nm, at which point the antioxidants present in the extracts and purified compounds began to inhibit the radical, producing a reduction in absorbance, with a quantitative relationship between the reduction and the concentration of antioxidants present in the texted sample. The radical scavenging activity is given as ABTS radical scavenging effect that is calculated by equation (2):

ABTS radical scavenging effect (%) = [(A_0_-A_1_)/A_0_] × 100 (2)

At the same time a standard curve was obtained using trolox standard solution at various concentrations (ranging from 0 to 100 μg/mL) in 95% ethanol. Scavenging activities of the purified compounds towards ABTS radical were expressed as TEAC (trolox equivalent antioxidant capacity). Different concentrations of each purified compound were chosen to test the ABTS radical scavenging activity. The results were compared with the standard curve for calculation of TEAC. Ascorbic acid (V_C_) and gallic acid were used for positive controls, respectively. All the tests were performed in triplicate.

#### 3.6.3. The free-radical-scavenging activities of the extracts and purified compounds were evaluated through Co (II) EDTA-induced luminol chemiluminescence by flow injection method

**Figure 4:**
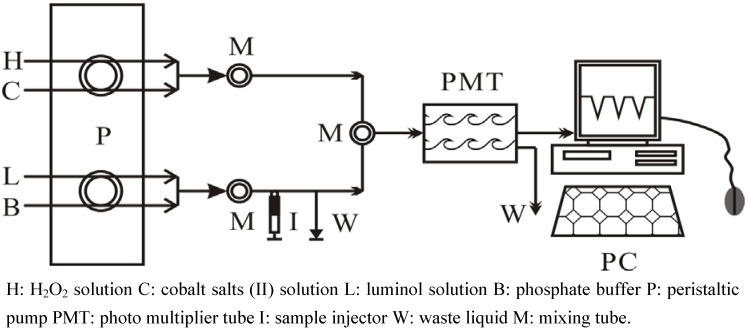
The schematic diagram of Flow-Injection Analysis apparatus.

Scavenging activities of the extracts and purified compounds from *M. paniculate* towards hydroxyl radical were assessed by Co (II) EDTA-induced luminol chemiluminescence by flow injection method described by Giokas, Vlessidis and Evmiridis with some modifications [25,26,27]. Briefly, the FIA (flow injection analysis) manifold was designed as shown in Figure 4. 

Once the pump was switched on, the Co (II)-stream (7.12 × 10^-4^ mol/L, pH = 9.0 ± 0.03), luminol-stream (2.28 × 10^-4^ mol/L, pH = 9.0 ± 0.03) and carrier-stream were initially mixed in order to reach a stable background. A stable baseline was obtained by mixing the H_2_O_2_-regent stream (0.8 × 10^-3^ mol/L, pH = 9.0 ± 0.03) into the system when cobalt (II) ion increased the chemiluminescene signal of luminol-H_2_O_2_ system. A loss of signal (negative peak) was observed which corresponded to the hydroxyl radical scavenging activity of the antioxidant when the antioxidant was injected to the system. Thus, the effect of antioxidant was measured by the depression of the signal from its initial level (uninhibited) and expressed as hydroxyl radical scavenging effect, calculated as follows:

Hydroxyl radical scavenging effect (%) = [height of negative peak/ (baseline-background)] × 100
(3)

Each extract or purified compound was injected three times on time intervals of at least 40 s. Ascorbic acid (V_C_) and gallic acid were used for positive controls, respectively. The scavenging activities of the purified compounds towards hydroxyl radical were also expressed as IC_50_.

### 3.7. Statistical analyses of results of activity studies

The results were performed as mean ± standard deviation (SD) of three determinations. Analysis of significance differences among means were tested by one-way analysis of variance. The IC_50_ values were calculated by linear regression analysis. Results were calculated by employing the statistical software (SPSS 13.0, SPSS Inc., USA.).

## 4. Conclusions

Free-radical-scavenging activities of four different solvent extracts (P.E., EtOAc, *n*-BuOH, and water) of *M. paniculata* stems were assessed through three *in vitro* model systems (DPPH, ABTS and Co (II) EDTA-induced luminol chemiluminescence by flow injection). It was found that the EtOAc extract displayed the highest free-radical-scavenging activity in all three assays, so it was fractioned chromatographically to yield five compounds **1-5** identified as a new triterpene named methyl 3*β*-*O*-*p*-hydroxy-*E*-cinnamoyloxy-2*α*,23-dihydroxy-olean-12-en-28-oate (**1**), together with four known compounds: epicatechin (**2**), 3-*trans*-feruloyl maslinic acid (**3**), maslinic acid (**4**) and sucrose (**5**). The structures of these compounds were elucidated by means of spectral data analysis and comparison with literature values. Free-radical-scavenging activities of the purified compounds (except compound **5**) were also measured with the three *in vitro* model systems above-mentioned, where trhey showed activities which decreased in the order of **2** > **3** > **1** > **4**. Among them, compound **2** with five hydroxyl groups exhibited the highest scavenging activity against DPPH and hydroxyl radicals with IC_50_ values of 2.83 μg /mL and 0.18 μg /mL, respectively (Table 1). The ABTS scavenging activity of **2** (TEAC = 1.38 ± 0.05) was found to significantly higher than that of Trolox (Table 1), and therefore it may be a promising molecule for food and medicinal applications. Further research on isolation and identification of more bioactive compounds from *M. paniculata* will be helpful to understand this traditional herbal medicine.
